# Using the Health Belief Model to Examine Parental Knowledge and Health Beliefs About Human Papilloma Virus (HPV) and iHPV Vaccine in Kuwait: Cross-Sectional Survey Study

**DOI:** 10.2196/75818

**Published:** 2025-12-09

**Authors:** Ahmad Abuzoor, Md Shafiqur Rahman Jabin, Cyril Eshareturi, Rae Nesbitt, Cor Jonker

**Affiliations:** 1 Department of Public Health, Faculty of Health Studies, University of Bradford, Bradford, United Kingdom; 2 Department of Medicine and Optometry, Linnaeus University, Pedalstråket 11, Kalmar, 392 31, Sweden, 46 (0)7915 673612; 3 Public Health Principal, London Borough of Ealing, London, United Kingdom; 4 Department of Public Health, Faculty of Health Studies, University of Bradford, Bradford, United Kingdom

**Keywords:** human papillomavirus, human papillomavirus vaccine, health belief model, parents.

## Abstract

**Background:**

Cervical cancer (CC) is a major public health issue, accounting for approximately 350,000 deaths, around 7.5% of all female cancer deaths worldwide, in 2018. Human papillomavirus (HPV) is the most common virus infecting the reproductive system. Despite the high number of diagnosed cases of CC globally, prevention is possible. Vaccination against HPV is considered to be a primary prevention strategy, while cervical screening can also play a secondary prevention role.

**Objective:**

This study aimed to examine the knowledge and health beliefs of parents in Kuwait towards HPV and HPV vaccination in order to prepare for the development of a national policy on CC.

**Methods:**

A cross-sectional survey was conducted among a representative multistage sample of 538 parents and guardians of eligible children aged 12–17 years in Kuwait, yielding a response rate of 89%. The survey was structured using the health belief model. Analysis showed statistically significant links between knowledge, health beliefs, concepts, and vaccination intention.

**Results:**

Knowledge of HPV and HPV vaccination was low in our study population: 55.6% (n=297), 24.9% (n=133), and 19.5% (n=104) for poor, fair, and good knowledge, respectively. Parents of daughters scored lower on perceived susceptibility to HPV and were more likely to have a higher perception of barriers to HPV vaccination, even though fathers were more likely to believe their daughters were at risk. HPV vaccination has the stigma of promiscuity attached, even though half of the parents are willing to accept HPV vaccination if that recommendation comes from Uhealth officials or relatives. A greater proportion of parents with female children had a low perception of the severity of HPV infection compared to those with male children (n=154, 58.6% vs n=134, 49.4%; *P*=.043). Around 52% (n=278) of parents perceived a high benefit of HPV vaccination. Parents with a female child had a lower perception of HPV vaccine benefits compared to parents with a male child. The findings demonstrated that parents with higher levels of education were better informed about the use of HPV vaccines in controlling the illness. Parents with female children were 1.34 times more likely to act on the recommendation for HPV vaccination compared to parents with male children after a recommendation from an official source, such as doctors or healthcare professionals.

**Conclusions:**

Recommendations for a Kuwaiti vaccination policy for HPV must take into consideration different knowledge levels of parents for groups with different educational levels, as well as the stigma of promiscuity and other barriers, and various health beliefs regarding susceptibility for daughters and sons, respectively.

## Introduction

Human papillomavirus (HPV), a sexually transmitted virus, accounts for 99% of cervical cancer (CC) cases worldwide [1]. Also, HPV is responsible for around 70% of vulvar and vaginal cancers and 60% of penile cancers. Approximately 80% of men and women are predicted to contract HPV over their lives, typically as a result of sexual activity [2]. The best prevention against HPV infection and, therefore, CC is the HPV vaccine. Getting HPV vaccination at younger ages is medically advantageous in various respects. HPV vaccines demonstrated over 99% effectiveness when given to women without previous HPV exposure [2]. There are over 100 species, with 30‐40 primarily found in the human genital tract. Sero-risk strains cause cervical, vulvar, vaginal, and anal cancers [3]. HPV 16 and 18 are implicated in 70% of all CC globally [4,5]. The vaccine is generally given in a series of 3 doses over 6 months to individuals aged 11 to 26, demonstrating considerable efficacy in preventing CC and decreasing the incidence of several HPV-related diseases [6]. In this study, a quantitative survey was conducted to evaluate parents’ knowledge and perceptions of HPV and its vaccination, aiming to determine demographic features of parents in Kuwait who have a child between the ages of 12 and 17 and parental awareness and health behaviors as measured by the health belief model (HBM), to create a comprehensive health promotion program for Kuwait.

Toward this end, the study has the following specific objectives: to determine Kuwaiti parents’ health beliefs and levels of knowledge on HPV and its vaccine. To examine health beliefs within the HBM about HPV and its vaccination. To evaluate the differences in health belief scores based on demographic characteristics and identify distinctions across different societal groups. To develop recommendations for health promotion to vaccinate against HPV. The lack of knowledge and awareness is a significant barrier to the uptake of HPV vaccination in Middle Eastern countries [7]. Previous research found higher acceptance rates among better-educated parents and those living in cities, which supports this pattern. To increase vaccination uptake in these populations, targeted interventions are required to meet the unique needs and concerns of rural and less educated populations [8].

Kuwait has lower rates of CC than other high-income nations like the United States and the United Kingdom, but its age-standardized death rate of 2.99 per 100,000 women is higher than the United States (2.77) and the United Kingdom (2.01). This gap in treatment and prevention highlights a significant gap in CC prevention. The rising prevalence of CC and deaths in Kuwait highlights the need for stronger preventive measures and better access to early detection and treatment services.

Mass vaccination of boys and girls could potentially eliminate HPV, the leading cause of CC, and contribute to the WHO’s universal campaign to end CC as a public health issue. However, recent research suggests that gender-neutral programs, including HPV shots for both genders, could be more effective. Gender norms may also affect vaccine acceptability, with females potentially facing greater challenges related to vaccination [9]. One study in Saudi Arabia mentioned that only 11% of parents were aware of HPV [10]. Another study in Turkey found that 24.5% of mothers and 21.2% of fathers were mindful of HPV vaccination [11]. In Iran, about 76% of parents were unaware of the seriousness of HPV infection. 76% of parents were unaware of the seriousness of HPV infection [12]. On the other hand, a high level of HPV vaccine knowledge was the most important factor influencing HPV vaccine acceptability, as evidenced by various studies. In a study of female university students studying in health sciences in Fujian Province, the results showed that the most important factor related to the intention to obtain the HPV vaccine was high HPV vaccination knowledge [13]. The most common ways of HPV vaccine promotion were hospital or school health education, as well as doctor or nurse recommendations [14].

The HPV vaccine is not part of Kuwait’s national vaccination program but is available in private medical care through the private health system [15]. Recently, at the end of 2024, it became optional in the health care center government. The absence of a national cervical screening program and HPV vaccination program is a significant obstacle to preventing CC in Kuwait. One of the essential motivations for this thesis is to reduce morbidity and mortality of CC by introducing the HPV vaccine (primary prevention) in the national immunization program in Kuwait. Also, the cost of treatment for cancer is more expensive than prevention by the HPV vaccine or early diagnosis by regular CC screening. New Kuwaiti studies on children’s vaccinations should consider parental perspectives before, during, and after HPV vaccination and integrate HPV vaccination into the national immunization program. Because vaccinations are given at a young age, parents are generally the primary decision makers, and their consent is required for immunization. Consequently, educational HPV campaigns have primarily concentrated on educating parents rather than children to enhance vaccination rates [16]

## Methods

### Overview

The cross-sectional descriptive questionnaire was conducted in Kuwait in May 2021, with 6 educational regions: the Asimah, Hawalli, Farwaniya, Ahmadi, Mubarak Al Kabeer, and Al-Jahra Governorates. The parents of individuals aged 12‐17 years in the intermediate stage in government schools have been chosen as eligible study participants. The sample size is calculated using a formula for estimating continuous data, as the study involves calculating knowledge and perception scores for health beliefs. The aggregate scores are measured through knowledge questions, where parents earn points for correct answers and have perception scores on a Likert scale. An 88-item questionnaire was designed for the study’s purpose. This consisted of 53 items based on HBM, 25 questions about HPV knowledge, the HPV vaccine, and HPV-associated cancers, and one additional question, “Would you like to participate in an interview?”

The study aimed to measure participants’ awareness of HPV and the HPV vaccine, as well as their health beliefs and experiences about vaccination. Closed-ended questions were used for statistical analysis, with each respondent given a choice of “True,” “False,” or “I do not know.” A 5-point Likert scale was used to measure parental perceptions and health beliefs, according to the HBM constructs.

The sample comprised equal numbers of boys and girls, with a sex ratio of 1:1. The CI is 95%, and the power is 80%. No previous studies in the literature have found anything that could help in predicting the scores. The sample size calculation is based on the perceived barriers score, a summation score ranging from 12 to 60 points. We assume that the means are 45 and 43 in the girls’ and boys’ groups, respectively. In addition, because no previous studies were found from which we could derive an SD, we are assuming an SD of 7 for each group, resulting in a sample size of 386. The margin of error is assumed to be 10%, and 35% of the questionnaires are expected to lack responses or be unreturned [17]. To compensate, the total sample size should be 573.21; rounding up, there will be 600 questionnaires—sample size calculation based on the Open Epi formula calculation [18].

A multistage sampling technique was used to select students for the study. In the first stage, 3 out of the 6 educational regions in Kuwait were chosen using simple random sampling. In the second stage, 20 schools were selected by ballot, without replacement, from the list of all schools in the region. An equal number of students (30 each) were recruited consecutively from each of the 20 schools in the final stage, for a total of 600 participants.

The questionnaire, originally developed in English, was then translated into Arabic, Kuwait’s official language, by a licensed translation center to ensure cultural and linguistic appropriateness for the target audience. This process was rigorous, designed to build an accessible, accurate data-collection instrument for the research goals. The questionnaire was administered in 2 stages: the pilot study and the final study. The survey was developed and presented in Arabic to participants. Before the pilot test, the researcher and supervisors reviewed the face validity of each item. The instrument was validated in a pilot test with 15 parents who did not participate in the final analysis. Following the pilot test, no further revisions were made to the questionnaire. The printed questionnaires and information sheets were given to students, who took them home to their parents and, upon completion, returned them to the schools, from which they were collected. During data collection, the vaccine was only available in private health sectors at a cost during the study period. Recently, at the end of 2024, it became optional in the government health care centers.

### Data Analysis

The study used a quantitative method to analyze the knowledge and beliefs about HPV vaccines among parents in Kuwait. The data was collected through questionnaires and coded, cleaned, and verified using SPSS version 27 software (IBM Corp). The data analysis was structured using questionnaire sections aligned with the HBM constructs. Descriptive statistics were used to represent the data clearly and easily, while graphs, tables, and statistical models were used to interpret and present numerical data [19].

The HBM scores were compared with demographic variables to discover patterns in responses. Descriptive and inferential statistics were used to compare trends in parental knowledge and health beliefs about the HPV vaccine. The descriptive analysis focused on central tendency and dispersion, while the statistical analysis aimed to predict behavioral effects associated with HPV vaccination uptake by finding meaningful patterns and correlations in the data.

The research question related to HBM was analyzed using a binary categorization of knowledge score (high or low) and HBM constructs based on the median. Both the Knowledge score and HBM variables were used in logistic regression to calculate the odds ratio [20]. Chi-square tests and logistic regression analyses were used to evaluate the relationships between categorical variables, such as parents’ interests in vaccinating their children against HPV [21]. Inferential analyses investigated the knowledge about the HPV vaccine and each HBM construct over different demographic groups. The *t* tests and ANOVA or Mann-Whitney *U* tests were applied to define mean and median scores between two or more groups.

Using unadjusted and adjusted odds ratios is shown to random imbalances. When using an adjusted method, there is a chance that the nature of the link between the covariate and outcome will be misspecified for continuous covariates. The disparities between parents, including age, sex, parent-child sex, and other participant variables, are disregarded. With these “baseline variables” adjusted, we can more accurately predict a response by taking these parent-to-parent differences into account. As a result, the analysis’s statistical power is increased. In conclusion, the quantitative method was chosen because it does not involve a direct relationship with participants and can improve response and decrease bias [22].

### Ethical Considerations

The study received ethical approval from the University of Bradford which had reference number (E843), the Ministry of Health in Kuwait with reference number (1345/2020), and the Ministry of Education in Kuwait with reference number (259006 and 00010918). Participants were informed about the research’s purpose, methodology, and confidentiality policy, ensuring anonymity and privacy. The researcher prioritized neutrality and objectivity for a credible data collection process. Participants signed informed consent forms, which explained the study’s purpose, methodology, and confidentiality policy. Parents provided informed consent for the pilot study. The questionnaire provided an explanation of the study’s purpose, methodology, and confidentiality policy. Participants indicated their consent by returning completed questionnaires, which were treated as implied consent. Data will be preserved for five years and archived within the University of Bradford, and no medical experiments, medicines, biopsies, or other interventional testing will be conducted in the future.

## Results

### Overview

**Figure 1.**
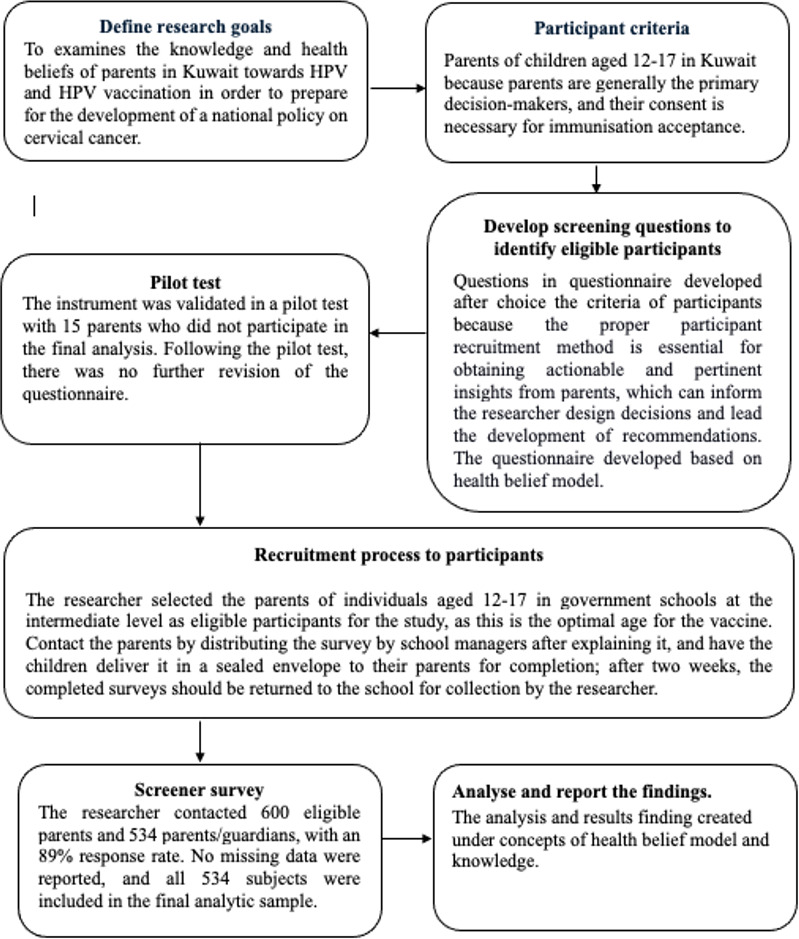
A flow diagram for research study recruitment process.

The researcher contacted 600 eligible parents and 534 parents and guardians, with an 89% response rate. No missing data were reported, and all 534 individuals were included in the final analytic sample. Figure 1 shows a flow diagram for the research study recruitment process.

### Demographic Characteristics

The study’s data quality in Kuwait indicates that the participants’ age is similar to the general population, with a mean age of 42.78 years (SD 5.71; range, 24‐60 y), identical to the average Kuwaiti age for marriage. The HPV vaccine is given to children aged 12‐16 years, considered the Intermediate School period in Kuwait. The mean age of children surveyed was 13.45 years (SD 1.23, range 12‐17 y), with almost as many boys as girls. Females filled in survey forms twice as many times as fathers, possibly due to the mother’s higher education and refusal to allow the father to fill in the form.

The employed versus unemployed ratios in Kuwait are similar to those of the general population, with many Kuwaitis participating. Almost half of the sampled population holds a Bachelor’s degree, similar to the general population. However, the females had higher education compared to males, which might be because educated females do not let the child’s father fill out the forms. Nearly half of the participants (n=247, 46.4%) have a monthly family income of 700-1500 KD. The data represent the Kuwaiti population well in all aspects, but there is evidence that females are slightly more educated than the general population, resulting in more participation in the survey.

### Previous Knowledge of HPV and Vaccine Among the Study Parents

The study found low knowledge of HPV and its vaccine among the population, with significant associations between demographic characteristics such as nationality, monthly family income, and educational level. Non-Kuwaiti parents of eligible children or adolescents had three times higher HPV knowledge compared to Kuwaitis. Parents with monthly family incomes of between US $2260 and US $4,800 were more likely to have a better understanding than those with lower incomes. Parents of eligible children with advanced degrees were 3.6 times more likely to have higher HPV knowledge.

### Health Belief Concepts

HBM builds on behavioral and psychological models that elaborate on two primary motivations for health behavior: (1) wanting to avoid illness or to recover from disease if already ill, and (2) believing that an action in the health world will prevent or cure illness. In most cases, a person makes a choice based on how they think about the advantages and disadvantages of health behavior.

### Perceived Susceptibility

The mean score of perceived susceptibility was 29.06 (SD 4.25), with scores ranging from 18.00 to 46.00. Female participants (n=363) outnumbered male participants (n=171) in the analysis (Multimedia Appendix 1).

Out of 534 participants, 12.0% (n=64) believed their adolescent sons would get anal cancer in their lifetime, compared to 12.9% (n=69) individuals who believed that their daughters would have CC. More men, compared to women, believed that their daughters were at risk of HPV during their lifetime (n=53, 31.0% vs n=66, 18.2%; *P*=.001; Multimedia Appendix 2)*.*

Statistically significant relationships were observed between perceived susceptibility to HPV infection and a number of sociodemographic characteristics of the study participants.

Table 1 showed a slightly greater proportion of participants with a bachelor’s degree had an increased perception of susceptibility to HPV compared to their counterparts of various educational levels (*P*<.001).

**Table 1.** Comparison of levels of perceived susceptibility (high vs low) versus demographic characteristics.

Demographic characteristics	Low (n=244)	High (n=290)	*P* value
Sex (Female), n (%)	140 (57.4)	148 (51.0)	.17
Parent sex (Male), n (%)	76 (31.1)	95 (32.8)	.76
Child age, mean (SD)	013.55 (1.27)	013.36 (1.18)	.06
Parent age, mean (SD)	43.07 (6.35)	42.54 (5.12)	.29
Income, n (%)			<.001^a^^b^
Less than 700 KD	41 (16.8)	30 (10.3)	
700‐1500 KD	132 (54.1)	117 (40.3)	
More than 1500 KD	71 (29.1)	143 (49.3)	
Marital status, n (%)			.005^a^^b^
Single	11 (4.5)	5 (1.7)	
Married	189 (77.5)	257 (88.6)	
Divorced	36 (14.8)	25 (8.6)	
Widowed	8 (3.3)	3 (1.0)	
Employment (unemployment), n (%)	28 (011.50)	31 (10.7)	.88
Nationality (non-Kuwait), n (%)	18 (7.4)	21 (7.2)	≥.99
Education, n (%)			<.001^a^^b^
Primary education	9 (3.7)	2 (0.7)	
High school education	52 (21.3)	31 (10.7)	
Diploma	53 (21.7)	64 (22.1)	
Bachelor’s degree	122 (50.0)	152 (52.4)	
Master’s or PhD	8 (3.3)	41 (14.1)	

a Indicates statistical significance.

b indicates the *χ*2 test.

Table 2 shows unadjusted and adjusted results for perceived susceptibility to HPV, categorized by a median score from our logistic regression models. In our unadjusted logistic regression models, participants’ educational level and monthly family income showed a statistically significant and positive correlation with perceived susceptibility to HPV.

**Table 2.** Logistic regression models illustrate the relationship between participants’ characteristics and their perceived susceptibility to human papillomavirus (HPV) infection among adolescents. Adjusted odds ratios are corrected for the other variables in the model and used for multivariate analysis, whereas unadjusted odds ratios are used for univariate analysis.

Characteristics	Perceived susceptibility to infection of HPV			
	Unadjusted odds ratio (95% CI)	*P* value	Adjusted odds ratio (95% CI)	*P* value
Age	0.880 (0.760‐1.010)	.06	0.870 (0.750‐1.020)	.82
Child adolescent sex				
Male (reference)	—^a^	—	—	—
Female	0.770 (0.550‐1.090)	.14	0.620 (0.420‐0.910)	.03^b^
Parent characteristics				
Age of respondent	0.98 (0.95‐1.01)	.29	0.99 (0.97‐1.03)	.55
Parent sex				
Female (reference)	—	—	—	—
Male	1.080 (0.750‐1.550)	.69	0.96 (0.62‐1.50)	.85
Nationality				
Kuwaiti (reference)	—	—	—	—
Non-Kuwaiti	0.98 (0.51‐1.90)	.95	0.97 (0.48‐1.99)	.94
Education				
Primary education (reference)	—	—	—	—
High school education	2.68 (0.64‐18.35)	.23	2.73 (0.57‐20.10)	.25
Diploma	5.43 (1.33‐36.67)	.04	6.06 (1.27‐44.69)	.04^b^
Bachelor’s degree	5.61 (1.41‐37.22)	.03	5.78 (1.23‐42.32)	.04^b^
Masters or PhD	23.06 (4.88‐171.98)	<.001^b^	21.31 (3.77‐178.35)	.001^b^
Monthly family income				
Less than 700 KD (reference)	—	—	—	—
700‐1500 KD	1.21 (0.71‐2.08)	.48	1.04 (0.58‐1.89)	.62
More than 1500 KD	2.75 (1.59‐4.80)	<.001^b^	1.73 (0.92‐3.26)	.91
Employment status				
Employment (reference)	—	—	—	—
Unemployment	0.920 (0.540‐1.590)	.77	1.780 (0.880‐3.700)	.74
Marital status				
Single (reference)	—	—	—	—
Married	2.99 (1.07‐9.63)	.05	2.06 (0.68‐7.05)	.22
Divorced	1.53 (0.49‐5.35)	.48	1.35 (0.40‐5.05)	.64
Widowed	0.83 (0.14‐4.43)	.82	0.42 (0.06‐2.49)	.34

a Not applicable.

b Indicates statistical significance.

Approximately half of the parents reported that HPV and its related diseases might affect their children. After adjusting for other parent and child or adolescent factors, significant associations were observed between education level, the child’s gender, and perceived susceptibility to HPV. Additionally, parents of a girl child did not perceive vulnerability (susceptibility) to HPV more readily than parents of a boy child.

### Perceived Severity

Multimedia Appendix 3 provides descriptive statistics for the overall perceived severity score of HPV across child participants, classified by gender. The study had more female (n=363) than male (n=171) participants. The perceived severity score of men and fathers was greater (mean 31.05, SD 9.53) than that of mothers and female guardians (mean 30.17, SD 9.24). The overall median perceived severity score for HPV was 30.46 (SD 9.34), ranging from 9.00 to 45.00.

A total of 27.2% (n=145) parents believed that infection with HPV might lead to morbidity (Multimedia Appendix 4). Although male and female participants generally held similar perceptions of HPV severity, many significant differences were observed. A higher percentage of men, compared to women, believed that HPV may result in serious health complications for their children. Moreover, a more significant number of men (n=88, 51.50%), compared to women (n=151, 41.60%; *P*=.04) believed that infection with HPV could have a negative impact on their children’s education, interruption of life (fathers: n=99, 57.90% vs mothers: n=165, 45.50%; *P*=.01), or impede their girls' chances of marriage (fathers: n=94, 55.00% vs mothers: n=144, 39.70%; *P*=.001).

We found statistically significant relationships between the perceived severity of HPV infection and a number of baseline sociodemographic characteristics of the study participants (Table 3). A more significant proportion of parents with female children (n=154, 58.6%) compared to male children (n=134, 49.4%; *P*=.04) had a low perception of the severity of HPV infection. Our analysis showed a significant percentage difference in perceived HPV infection severity among respondents of non-Kuwaiti nationality (low severity: n=9, 3.4% vs high severity: n=30, 11.1%; *P*=.001).

**Table 3.** Comparisons of levels of perceived severity versus demographic characteristics.

Demographic characteristics	Low (n=263)	High (n=271)	*P* value^a^
Sex (Female), n (%)	154 (58.6)	134 (49.4)	.04^b^
Sex of parent (Male), n (%)	75 (28.5)	96 (35.4)	.11
Child age, mean (SD)	013.42 (1.19)	013.47 (1.26)	.66
Parents’ age, mean (SD)	43.47 (5.52)	042.11 (5.82)	.006^b^
Income monthly, n (%)			.61
Under 700 KD	37 (14.1)	34 (12.5)	
700‐1500 KD	117 (44.5)	132 (48.7)	
More than 1500 KD	109 (41.4)	105 (38.7)	
Marital status, n (%)			.32
Single	6 (2.30)	10 (3.70)	
Married	215 (81.7)	231 (85.2)	
Divorced	36 (13.7)	25 (9.2)	
Widowed	6 (2.30)	5 (1.80)	
Employment (unemployment), n (%)	30 (11.4)	29 (10.7)	.90
Nationality (non-Kuwait), n (%)	9 (3.4)	30 (11.1)	.001^b^
Education, n (%)			.054^a^
Primary education	9 (3.4)	2 (0.7)	
High school education	39 (14.8)	44 (16.2)	
Diploma	59 (22.4)	58 (21.4)	
Bachelor’s degree	139 (52.9)	135 (49.8)	
Masters or PhD	17 (6.5)	32 (11.8)	

a indicates the *χ*2 test.

b Indicates statistical significance.

Table 4 shows unadjusted and adjusted results for the perceived severity of HPV, categorized by median severity score from our logistic regression models. In the unadjusted logistic regressions, child sex (female), age, education, and nationality were statistically associated with perceived HPV severity. Parents of a female child had 29% less empathy for the severity of HPV than parents of a male child (OR: 0.69, 95% CI 0.49‐0.97, *P*=.04). For each year of age increase, participants perceived 4% less severe HPV infection (OR: 0.96, 95% CI 0.93‐0.99, *P*=.006).

**Table 4.** The association between the characteristics of participants and their perceived severity of infection of human papillomavirus (HPV) among adolescents is illustrated by logistic regression models.

Characteristics of adolescents	Perceived severity of the infection of HPV			
	Unadjusted odds ratio (95% CI)	*P* value	Adjusted odds ratio (95% CI)	*P* value
Child age	1.03 (0.89‐1.19)	.66	1.09 (0.94‐1.27)	.23
Child adolescent sex				
Male (reference)	—^a^	—	—	—
Female	0.69 (0.49‐0.97)	.04^b^	0.67 (0.46‐0.98)	.04^b^
Parent characteristics				
Age of respondent	0.96 (0.93‐0.99)	.006^b^	0.95 (0.92‐0.98)	.004
Parent sex				
Female (Reference)	—	—	—	—
Male	1.38 (0.96‐1.99)	.09	1.30 (0.85‐1.99)	.23
Nationality				
Kuwaiti (reference)	—	—	—	—
Non-Kuwaiti	3.51 (1.70‐7.99)	.001^b^	3.61 (1.67‐8.52)	.002^b^
Education				
Primary education (reference)	—	—	—	—
High school education	5.08 (1.22‐34.65)	.05^b^	3.89 (0.82‐28.44)	.12
Diploma	4.42 (1.08‐29.84)	.06	3.74 (0.81‐27.09)	.12
Bachelor’s degree	4.37 (1.10‐29.01)	.06	4.28 (0.94‐30.63)	.09
Masters or PhD	8.47 (1.92‐59.87)	.01^b^	7.89 (1.54‐61.01)	.02^b^
Monthly household income				
Less than US $2260	—	—	—	—
Income US $2260-4800	1.23 (0.72‐2.09)	.45	1.32 (0.74‐2.38)	.35
More than US $4800	1.05 (0.61‐1.79)	.86	0.97 (0.52‐1.81)	.93
Employment status				
Employment (Reference)	—	—	—	—
Unemployment	0.93 (0.540‐1.60)	.79	1.14 (0.59‐2.21)	.69
Marital status				
Single (Reference)	—	—	—	—
Married	0.65 (0.22‐1.77)	.40	0.64 (0.20‐1.87)	.42
Divorced	0.42 (0.13‐1.27)	.13	0.44 (0.12‐1.44)	.18
Widowed	0.50 (0.09‐2.36)	.38	0.53 (0.10‐2.65)	.44

a Not appilcable.

b Indicates statistical significance.

In total, 50% (n=267) of the children’s parents had a high perception of HPV severity. A greater proportion of parents with female children had a low perception of the severity of HPV infection (n=154, 58.6% vs n=134 , 49.4%; *P*=.04), compared to those with male children. We also found that Kuwaiti nationals, parents of male children, and parents of younger children had significantly higher odds of reporting high perceived severity of HPV. This suggests that these groups may have more awareness or concern about the potential consequences of HPV infection.

### Perceived Benefits

Multimedia Appendix 5 presents the descriptive statistics for the perceived benefit scores of HPV vaccination among child parents, categorized by gender. The analysis includes more female participants (n=363) than male participants (n=171). Fathers and male guardians had a slightly higher mean of the total benefit score (mean 28.96, SD 8.57) than mothers and female guardians (mean 27.41, SD 8.01). These data reveal a slight difference in perceptions of HPV vaccine benefits for male and female guardians. In the sample, 51% of parents recognized HPV vaccination as a significant means for preventing cervical and anal cancer. The overall mean perceived score of HPV was mean 27.9 (SD 8.22, range: 8.00‐40.00).

Multimedia Appendix 6 showed that more than half of the parents believed that preventing cancer diseases by HPV vaccination is better than curing them, 51.8% (n=282). However, less than half (44.4%) believed the HPV vaccine is successful in preventing HPV infection for their daughters or sons. Some items of the perceived benefit of the HPV vaccine differed by parent sex. Differences were also noted in comparison to male parents. For instance, a smaller percentage of female parents agree that an HPV vaccine for their sons would help them stop worrying about anal and penile cancer compared to their male counterparts. Furthermore, a much smaller proportion of female parents felt the HPV vaccine was safe and prevented disease compared to their male counterparts.

The researcher found statistically significant relationships between the perceived benefit of the HPV vaccine or vaccination and a number of baseline sociodemographic characteristics of the study participants (refer to Table 5). The perceived benefit of the HPV vaccine or vaccination differed according to parents’ sex and level of education. There was a greater proportion of male respondents with a high perception of HPV vaccine or vaccination benefits than females (n=69, 26.8% vs n=102, 36.8%; *P*=.02). However, there were more respondents with a bachelor’s degree among those in the group with a high perception of the benefits of the HPV vaccine compared with those with a lower perception of benefit (n=133, 51.8% vs n=141, 50.9%; *P*=.02).

**Table 5.** Comparison between perceived benefits of HPV vaccination and demographic characteristics.

Demographic characteristics	Low (n=257)	High (n=277)	*P* value
Sex (Female), n (%)	148 (57.6)	140 (50.5)	.12
Parent sex (Male), n (%)	69 (26.8)	102 (36.8)	.02^a^
Child age, mean (SD)	13.42 (01.20)	13.47 (01.25)	.59
Parent age, mean (SD)	42.34 (05.27)	43.19 (6.08)	.09
Income, n (%)			.07
Less than US $2260	43 (16.7)	28 (10.1)	
US $2260‐4800	118 (45.9)	131 (47.3)	
More than US $4800	96 (37.4)	118 (42.6)	
Marital status, n (%)			.21
Single	4 (1.6)	12 (4.3)	
Married	214 (83.3)	232 (83.8)	
Divorced	33 (12.8)	28 (10.1)	
Widowed	6 (2.30)	5 (1.80)	
Employment unemployment, n (%)	27 (10.50)	32 (11.60)	.80
Nationality (non-Kuwait), n (%)	16 (6.20)	23 (8.30)	.45
Education, n (%)			.02^a^
Primary education	9 (3.5)	2 (0.7)	
High school education	40 (15.6)	43 (15.5)	
Diploma	60 (23.3)	57 (20.6)	
Bachelor’s degree	133 (51.8)	141 (50.9)	
Master’s or PhD	15 (5.8)	34 (12.3)	

a Indicates statistical significance.

An independent *t* test—Test of Levene’s (*P* >.05) was not significant; hence, equal variances are assumed for the independent *t* test—The results, categorized by gender based on total perceived benefit scores, were presented in Multimedia Appendix 7. This test has a *P* value less than the α-value of .05, so there is sufficient data to infer a significant difference between the benefit scores of male (fathers and male guardians) and female (mothers and female guardians) individuals (*t*_532_=-2.045; *P*=.04).

Table 6 demonstrates unadjusted and adjusted results for the perceived benefit of HPV, categorized by median benefit score from logistic regression models.

**Table 6.** Logistic regression models illustrate the relationship between participants’ characteristics and their perceived benefit from human papillomavirus (HPV) infection among adolescents.

Characteristics of adolescents	Perceived benefit of HPV vaccine or vaccination			
	Unadjusted odds ratio (95% CI)	*P* value	Adjusted odds ratio (95% CI)	*P* value
Child age	1.04 (0.90‐1.19)	.59	1.04 (0.89‐01.21)	.57
Child or adolescent sex				
Male (reference)	—^a^	—	—	—
Female	0.75 (0.53‐1.06)	.10	0.68 (0.47‐0.99)	.04
Parent characteristics				
Age of respondent	1.03 (0.99‐1.06)	.09	1.02 (0.99‐1.06)	.17
Parent sex				
Female (reference)	—	—	—	—
Male	1.59 (1.100‐02.30)	.01	1.37 (0.89‐02.09)	.15
Nationality				
Kuwaiti (reference)	—	—	—	—
Non-Kuwaiti	1.36 (0.71‐2.69)	.36	1.52 (0.75‐3.15)	.25
Education				
Primary education (reference)	—	—	—	—
High school education	4.84 (1.16‐33.01)	.05	3.50 (0.73‐25.84)	.15
Diploma	4.28 (1.05‐28.84)	.07	4.41 (0.93‐32.55)	.09
Bachelor’s degree	4.77 (1.20‐31.67)	.05	4.96 (1.06‐36.28)	.06
Masters or PhD	10.20 (2.29‐72.48)	.006	9.47 (1.80‐74.75)	.01
Monthly family income				
Less than 700 KD (reference)	—	—	—	—
700‐1500 KD	1.70 (1.00‐2.94)	.05	1.66 (0.92‐3.03)	.09
More than 1500 KD	1.89 (1.09‐3.29)	.02	1.65 (0.88‐3.13)	.12
Employment status				
Employment (reference)	—	—	—	—
Unemployment	1.11 (0.65‐01.93)	.70	01.41 (0.72‐2.78)	.32
Marital status				
Single (reference)	—	—	—	—
Married	0.36 (0.10‐1.06)	.08	0.27 (0.07‐0.84)	.03
Divorced	0.28 (0.07‐0.91)	.05	0.256 (0.06‐0.89)	.04
Widowed	0.28 (0.05‐1.38)	.13	0.17 (0.03‐0.89)	.04

a Not applicable.

Approximately 52.0% (n=278) of parents perceived a high benefit of HPV vaccination. Parents with a female child demonstrated a lesser perception of the benefits of the HPV vaccine compared to those with a male child. Additionally, higher levels of parental education were strongly associated with greater perceived benefits.

### Perceived Barriers

About 72.0% (n=388) of parents perceived that there are barriers to HPV vaccination for cervical and anal cancer prevention. The mean score for the perceived barrier to HPV vaccination was 37.13, SD 8.22 (range: 12.00‐60.00; Multimedia Appendix 8).

In Multimedia Appendix 9, up to 20% of parents who completed the survey believed that the stigma associated with receiving HPV vaccination and the perception that receiving the HPV vaccine is synonymous with promiscuity, according to the Kuwaiti culture, are key barriers to the uptake of the vaccine. Also, only 13%‐28% of participants acknowledged restrictions from their religion, lack of vaccine availability in public hospitals, affordability, unwillingness to pay for the vaccines, or fear of needles on the part of children as barriers to HPV vaccine uptake. There were no statistically significant differences in responses between male and female participants on these statements. However, they differed on the need for reassurance about the effectiveness and safety of the HPV vaccine. More males thought they needed reassurance about effectiveness (*P*=.006) and safety (*P*=.001).

Perceived barriers to the HPV vaccine or vaccination differed by sex of child, employment status, monthly family income, and level of education (see Table 7). We found a more significant proportion of parents with female children with the higher perception of barriers to the HPV vaccine or vaccination than those with male children (n=67, 45.9 vs n=221, 57.0; *P*=.03). Similar trends were observed for family income; monthly family income significantly differed between participants with high versus low perceived barriers to HPV infection (*P*<.01). Since the *P* value was more significant than the significance level α-value of 0.05, it can be concluded that there is no statistically significant difference in the barrier scores between male participants (fathers and male guardians) and female participants (mothers and female guardians). The results of the test were *t*_532_=–1.362; *P*=.17. This suggests that gender does not significantly influence the perceived barriers related to HPV vaccination.

**Table 7.** Comparison between perceived barriers and demographic characteristics.

Demographic characteristics	Low (n=146)	High (n=388)	*P* value^a^
Sex (Female), n (%)	67 (45.9)	221 (57.0)	.03^b^
Parent sex (Male), n (%)	38 (26.0)	133 (34.3)	.09
Child age, mean (SD)	013.48 (1.21)	013.43 (1.230)	.70
Parent age, mean (SD)	64 (6.45)	84 (05.42)	.72
Monthly family income, n (%)			.01^b^
Under 700 KD	28 (19.2)	43 (11.1)	
700‐1500 KD	71 (48.6)	178 (45.9)	
More than 1500 KD	47 (32.2)	167 (43.0)	
Marital status, n (%)			<.001^b^
Single	12 (8.2)	4 (1.0)	
Married	99 (67.8)	347 (89.4)	
Divorced	27 (18.5)	34 (8.8)	
Widowed	8 (5.5)	3 (0.8)	
Employment (unemployed), n (%)	29 (19.9)	30 (7.7)	<.001^b^
Nationality (non-Kuwaiti), n (%)	7 (4.8)	32 (8.2)	.24
Education, n (%)			<.001^b^
Primary education	7 (4.8)	4 (1.0)	
High school education	38 (26.0)	45 (11.6)	
Diploma	26 (17.8)	91 (23.5)	
Bachelor’s degree	64 (43.8)	210 (54.1)	
Master’s or PhD	11 (7.5)	38 (9.8)	

a indicates *χ*2 test.

b Indicates statistical significance.

Table 8 demonstrates unadjusted and adjusted results for a perceived barrier of HPV from our logistic regression models. The sex of the child (female), parents’ level of education, employment status, monthly family income, and marital status demonstrated a statistically significant correlation with a perceived barrier to HPV vaccination in the unadjusted logistic regression models. Parents with female children are likely to have a 1.6 times higher perception of the barrier to HPV vaccination compared to parents with male children (OR: 1.56, 95% CI 1.07‐2.29; *P*=.02).

**Table 8.** Logistic regression models illustrate the relationship between participants’ characteristics and their perceived barriers to human papillomavirus (HPV) infection among adolescents.

Characteristics of adolescents	Perceived barriers to HPV vaccination			
	Unadjusted odds ratio (95% CI)	*P* value	Adjusted odds ratio (95% CI)	*P* value
Child age	0.97 (0.83‐01.13)	.70	0.98 (0.82‐01.168)	.82
Child or adolescent sex				
Male (reference)				
Female	1.560 (1.070‐02.29)	.02^a^	1.60 (1.03‐2.47)	.04^a^
Parent characteristics				
Age of respondent	1.01 (0.97‐1.04)	.72	1.01 (0.97‐1.05)	.55
Parent sex				
Sex female (reference)				
Sex male	1.48 (0.98‐2.29)	.06	1.89 (1.13‐3.21)	.02^a^
Nationality				
Kuwaiti (reference)				
Non-Kuwaiti	1.78 (0.81‐4.49)	.18	1.69 (0.70‐4.67)	.27
Education				
Primary education (reference)				
High school education	2.07 (0.58‐8.42)	.27	0.80 (0.18‐3.74)	.77
Diploma	6.13 (1.72‐24.93)	.006^a^	2.69 (0.63‐12.57)	.19
Bachelor’s degree	5.74 (1.68‐22.51)	.007^a^	2.05 (0.49‐9.31)	.32
Masters or PhD	6.05 (1.55‐26.91)	.01^a^	2.15 (0.45‐11.22)	.34
Monthly family income				
Less than 700 KD (reference)				
700‐1500 KD	1.63 (0.94‐2.82)	.08^a^	1.18 (0.60‐2.26)	.62
More than 1500 KD	2.31 (1.30‐4.11)	.004^a^	1.04 (0.50‐2.11)	.91
Status of employment				
Employment (reference)				
Unemployment	0.34 (0.190‐0.59)	<.001^a^	0.89 (0.44‐1.83)	.74
Marital status				
Single (reference)				
Married	10.52 (3.58‐38.27)	<.001^a^	9.39 (2.86‐37.50)	<.001^a^
Divorced	3.78 (1.17‐14.74)	.04^a^	3.94 (1.07‐17.12)	.04^a^
Widowed	1.13 (0.18‐6.52)	.89	1.03 (0.15‐6.72)	.97

a Indicates statistical significance.

More than two-thirds of parents who responded to the survey alluded to barriers to HPV vaccination. The sex of the child and marital status are the factors that demonstrated significant associations with perceived barriers to HPV vaccination.

### Perceived Self-Effect

Multimedia Appendix 10 provides descriptive statistics for the overall perceived self-efficacy score related to HPV among child participants. The mean self-efficacy score for all individuals was 23.51 (SD 6.03). The study had more women (n=363) than men (n=171). Dads and male caregivers scored higher on the overall self-efficacy score (mean 25.09, SD 5.32) than moms and female caregivers (mean 22.77, SD 6.21).

Multimedia Appendix 11 shows that more than 40% of parents affirmed complete control over giving their son or daughter the HPV vaccine against anal, penile, or CC. However, about 33.7% (n=180) had good enough knowledge to decide on HPV vaccine uptake for their children. In addition, less than 40% (n=210) felt they could do something about their health by getting vaccinated against HPV. More than 40% (n=210) of parents affirmed complete control over giving their son or daughter HPV vaccine against anal, penile, or CC. However, about 33.7% (n=180) had good enough knowledge to decide on HPV vaccine uptake for their children. In addition, less than 40% (n=210) felt they could do something about their health by getting vaccinated against HPV.

Table 9 shows that parents with female children had a slightly greater percentage of high perceived self-efficacy than those with low self-efficacy (n=154, 59.0% vs n=134, 49.1%; *P*=.03). We found a greater proportion of male respondents with a high perception of perceived self-efficacy than females (n=58, 22.2 % vs n=113, 41.1%;, *P*<.001). Our analysis revealed a significant percentage difference in perceived self-efficacy among respondents of non-Kuwaiti nationality (n=11, 4.2% vs n=28, 10.3%; *P*=.01).

**Table 9.** Self-efficacy and baseline characteristics of respondents and children.

Demographic characteristics	Low (n=261)	High (n=273)	*P* value^a^
Sex of child, (Female), n (%)	154 (59.00)	134 (49.10)	.03^b^
Sex of parent, (Male), n (%)	58 (22.20)	113 (41.40)	<.001^b^
Child age, mean (SD)	13.47 (1.230)	13.42 (1.230)	.64
Respondent age, mean (SD)	42.57 (5.730)	42.99 (5.700)	.40
Income monthly, n (%)			.08
Under US $ 2260 $	36 (13.80)	35 (12.80)	
Between US $2260 and US $4800	133 (51.00)	116 (42.50)	
Over US $4800	92 (35.20)	122 (44.70)	
Marital status, n (%)			.20
Unmarried	6 (2.30)	10 (3.70)	
Wedded	212 (81.20)	234 (85.70)	
Divorce	37 (4.20)	24 (8.80)	
Widow	6 (2.30)	5 (1.80)	
Employment (unemployment), n (%)	30 (11.50)	29 (10.60)	.86
Nationality (non-Kuwaiti), n (%)	11 (4.20)	28 (10.30)	.01^b^
Level of education, n (%)			.001^b^
Primary education	11 (4.20)	0 (0.00)	
High school education	42 (6.1)	41 (15.0)	
Diploma	57 (21.8)	60 (22.0)	
Bachelor’s degree	136 (52.1)	138 (50.5)	
Masters or PhD	15 (5.7)	34 (12.5)	

a indicates *χ*2 test.

b Indicates statistical significance.

Unadjusted logistic regression models, the sex of the parent (male) was found to have a 2.47 times higher perception of self-efficacy for HPV vaccination compared to female parents (OR: 2.47; 95% CI (1.70‐3.63), *P*<.001). Adjusted models showed a significant association of sex of parent (male, 2.23; 95% CI (1.49‐3.39), *P*<.001). Nationality with perceived self-efficacy to HPV vaccination in unadjusted logistic regression models showed significant predictors of perceived self-efficacy of HPV vaccination (2.60; 95% CI 1.29‐5.55, *P*=.009), and in adjusted logistic regression models showed (3.11; 95% CI 1.50‐6.86, *P*=.003) (Table 10)

**Table 10.** Logistic regression models illustrate the relationship between participants' characteristics and their perceived self-efficacy of human papillomavirus (HPV) vaccination to prevent HPV infection and its consequences among children.

Characteristics of adolescents	Perceived self-efficacy of vaccination to prevent HPV infection			
	Unadjusted odds ratio (95% CI)	*P* values	Adjusted odds ratio (95% CI)	*P* values
Child age	0.97 (0.84‐01.11)	.64	0.98 (0.85‐01.14)	.83
Child or adolescent age				
Male (Reference)	—^a^	—	—	—
Female	0.67 (0.48‐0.94)	.02	0.69 (0.47‐0.99)	.04
Parent characteristics				
Age of respondent	1.01 (0.98‐1.04)	.39	1.00 (0.97‐1.04)	.77
Parent sex				
Sex female (reference)	—	—	—	—
Sex male	2.47 (1.70‐3.63)	<.001	2.23 (1.49‐3.39)	<.001
Nationality				
Kuwaiti (reference)	—	—	—	—
Non-Kuwaiti	2.60 (1.29‐5.55)	.009	3.11 (1.50‐6.86)	.003
Monthly family income				
Less than US $2260 (reference)	—	—	—	—
US $2260‐4800	0.89 (0.53‐1.53)	.69	0.90 (0.51‐1.59)	.72
More than US $4800	1.36 (0.79‐2.34)	.26	1.39 (0.77‐2.51)	.28
Employment status				
Employment (reference)	—	—	—	—
Unemployment	0.920 (0.53‐01.57)	.75	01.09 (0.59‐1.98)	.78
Marital status				
Single (reference)	—	—	—	—
Married	0.66 (0.22‐1.81)	.43	0.57 (0.18‐1.67)	.32
Divorced	0.39 (0.12‐1.19)	.10	0.42 (0.13‐1.34)	.15
Widowed	0.50 (0.10‐2.36)	.38	0.46 (0.09‐2.29)	.34

a Not applicable.

There was a significant difference in the perceived self-efficacy scores for fathers and male guardians and the mothers and female guardians, which means that males have more confidence in themselves as compared to females. The sex of the child (son for focus more than female daughter) and nationality were the significant predictors of perceived self-efficacy to HPV vaccination.

### Cue to Action

Multimedia Appendix 12 provides descriptive statistics for the total cue-to-action score related to HPV vaccination with the parents of children. The overall mean score for perceived self-efficacy of the HPV vaccine was 23.34 (SD=7.19). This study was conducted with more women (n=363) and men (n=171). Fathers and male guardians scored less cue-to-action on average (mean 22.54, SD 7.32) than mothers and female guardians, with a higher mean score (mean 23.93, SD 7.07).

Multimedia Appendix 13 shows that up to 50% of parents believed that stimuli such as recommendations from health professionals, relatives, and health promotion by MOH would trigger the decision-making process to accept HPV vaccination for their children. More than half of respondents said they would get vaccinated if health professionals recommended the HPV vaccine for their children.

Parents’ likelihood to cue to action for deciding to take the HPV vaccine differed by the child’s sex, nationality, marital status, and education (Table 11). We found a slightly more significant percentage of parents with female children with a higher perception of cue to action for HPV vaccination than those with a lower perception of cue to action (n=123, 48.6% vs n=165, 58.7%; *P*=.02). Married participants were more likely to take action against HPV vaccination than participants with other marital statuses (*P*=.04).

**Table 11.** Comparison between chances to cue to action for human papillomavirus (HPV) vaccination and demographic characteristics.

Demographic characteristics	Low (n=253)	High (n=281)	*P* value^a^
Child sex (female), n (%)	123 (48.6)	165 (58.7)	.02^b^
Parent sex (Male), n (%)	86 (34.0)	85 (30.2)	<.001^b^
Child age, mean (SD)	13.51 (0.20)	13.39 (01.25)	.25
Parent age, mean (SD)	42.83 (05.28)	42.74 (6.09)	.85
Income monthly, n (%)			.68
Under US $2260	32 (12.6)	39 (13.9)	
US $2260‐4800	123 (48.6)	126 (44.8)	
More than US $4800	98 (38.7)	116 (41.3)	
Marital status, n (%)			.04^b^
Single	4 (1.6)	12 (4.3)	
Married	210 (83.0)	236 (84.0)	
Divorced	36 (14.2)	25 (8.9)	
Widowed	3 (1.2)	8 (2.8)	
Employment (unemployed), n (%)	28 (11.1)	31 (11.0)	1
Nationality (non-Kuwait), n (%)	11 (4.3)	28 (10.0)	.02^b^
Education level (%)			<.001^b^
Primary education	9 (3.6)	2 (0.7)	
High school education	50 (19.8)	33 (11.7)	
Diploma	52 (20.6)	65 (23.1)	
Bachelor’s degree	130 (51.4)	144 (51.2)	
Masters or PhD	12 (4.7)	37 (13.2)	

a indicates the *χ*2 test.

b Indicates statistical significance.

Table 12 demonstrates unadjusted and adjusted results related to the likelihood of cue-to-action for uptake of HPV vaccination, categorized by a median cue-to-action score from logistic regression models. Our unadjusted logistic regression models showed that the sex of the child (female), level of education, nationality, and marital status demonstrated a statistically significant correlation with a child’s (female), level of education, nationality, and marital status demonstrated a statistically significant correlation with a perceived cue to action to HPV vaccination uptake. Parents with female children were 1.5 times more likely to cue to action for HPV vaccination than parents with male children (OR: 1.50; 95% CI 1.07‐2.12, *P*=.02). Similar trends of positive correlation were observed in the analysis. We found a significant increase in likelihood to cue to action for uptake of HPV vaccination for each step of education achieved among respondents compared to primary education (High School; OR: 4.84, 95% CI 1.16‐33.01, *P*=.05; Diploma; OR: 5.63; 95%CI 1.38‐37.96, *P*=.03; Bachelor’s Degree; OR: 4.98; 95%CI 1.26‐33.09, *P*=.04; Masters or PhD; 13.88 3.07‐99.86, *P*=.002). Married respondents were less likely to cue to action for HPV vaccination uptake for their children and themselves (OR: 0.23; 95% CI 0.06‐0.75, *P*=.02). Respondents of non-Kuwaiti nationality were more likely to cue to action for HPV vaccination uptake compared to Kuwaiti (OR: 2.43; 95% CI 1.22‐5.21, *P*=.02).

**Table 12.** Logistic regression models illustrate the relationship between participants' characteristics and their cue to action regarding human papillomavirus vaccination in children.

Characteristics of adolescent	Cue to action			
	Unadjusted odds ratio (95% CI)	*P* values	Adjusted odds ratio (95% CI)	*P* values
Child age	0.92 (0.80‐01.06)	.25	0.92 (0.79‐01.07)	.27
Child or adolescent sex				
Male (Reference)	—	—	—	—
Female	1.50 (1.07‐2.12)	.02^a^	1.34 (0.92‐1.95)	.13
Parent characteristics				
Age of respondent	0.99 (0.97‐1.03)	.85	1.01 (0.98‐1.05)	.41
Parent sex				
Female (reference)	—	—		—
—— Male.	0.84 (0.58‐01.21)	.36	0.87 (0.57‐01.35)	.54
Nationality				
Kuwaiti (reference)	—	—	—	—
Non-Kuwaiti	2.43 (1.22‐5.21)	.015^a^	1.90 (0.91‐4.20)	.01^a^
Education				
Primary education (reference)	—	—	—	—
High school education	2.97 (0.71‐20.29)	.18	2.08 (0.44‐15.29)	.39
Diploma	5.63 (1.38‐37.96)	.03^a^	5.06 (1.09‐36.79)	.05^a^
Bachelor’s degree	4.98 (1.26‐33.09)	.04^a^	4.65 (1.02‐33.43)	.03^a^
Masters or PhD	13.88 (3.07‐99.86)	.002^a^	15.58 (2.99‐122.55)	.003^a^
Monthly family income				
Less than US $2260 (reference)				
US $2260‐4800	0.84 (0.49‐1.43)	.52	0.62 (0.34‐1.12)	.12
More than US $4800	0.97 (0.56‐1.66)	.92	0.51 (0.27‐0.95)	.04^a^
Employment status				
Employment (reference)	—	—	—	—
Unemployment	0.99 (0.58‐1.72)	.99	1.26 (0.64‐2.50)	.51
Marital status				
Single (reference)	—	—	—	—
Married	0.37 (0.10‐1.09)	.09	0.33 (0.09‐1.03)	.07
Divorced	0.23 (0.06‐0.75)	.02^a^	0.20 (0.05‐0.69)	.02^a^
Widowed	0.89 (0.15‐5.54)	.89	0.74 (0.12‐4.82)	.74

a Indicates statistical significance.

The researcher observed significant correlations between level of education and marital status and respondents’ likelihood of acting on the cue to take up HPV vaccination. About 53% (n=283) of parents were more likely to act on the recommendation for HPV vaccination uptake after a recommendation from health care professionals.

### Correlation Between Participants and Knowledge

**Figure 2.**
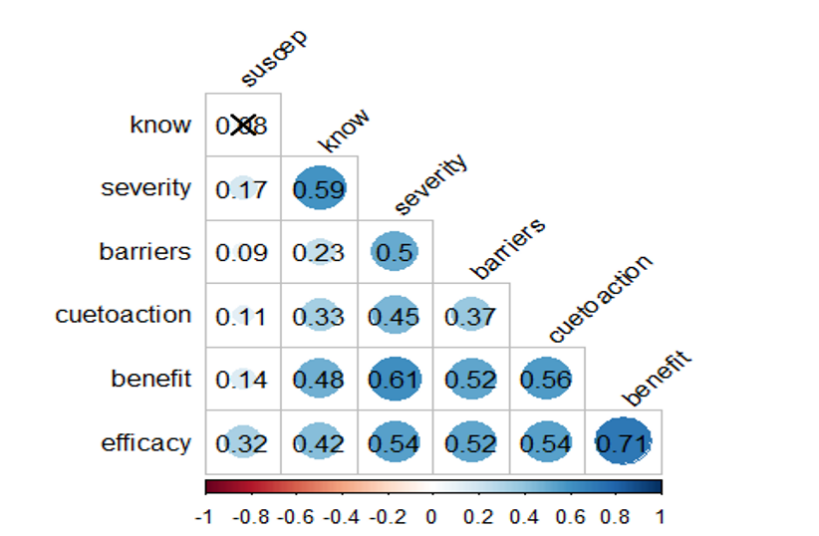
The matrix of correlation between the total knowledge score and the overall scores for each of the six HBM components.

There was a positive correlation between all 6 constructs and knowledge of HPV, as the *P* value for all the correlation coefficients was less than 0.05. As one variable increases, the other also increases, and the degree of the relationship was statistically significant for all correlations except for susceptibility to HPV and knowledge of HPV. The strongest correlation was seen between the benefit of HPV vaccination and self-efficacy to recommend or make decisions for HPV vaccination for children (*r*=0.71, *P*<.001). The significant but weakest correlation was barriers to HPV vaccination and perceived susceptibility to HPV (*r*=0.09, *P*<.05), as seen in Figure 2.

## Discussion

### Principal Findings

The survey findings significantly contribute to the study’s overall aim by providing insights into the factors that affect parents’ acceptance of HPV vaccination in Middle Eastern countries. It is pertinent to mention that although the aim of the current thesis was specific to the State of Kuwait. The researcher targeted available literature for all Middle Eastern countries because Kuwait and other Middle Eastern countries share several common characteristics.

Our findings were consistent with other research using the HBM to assess the level of knowledge, beliefs, and acceptability of the HPV vaccine among parents of their children. For instance, one study stated that Kuwaiti university students were lagging in their knowledge of HPV and the HPV vaccine. In addition, the research reported that vaccine conspiracy beliefs were negatively correlated with their willingness to receive vaccination, demonstrating the role of misinformation and beliefs in decision-making around vaccination [23]. These findings agreed with our observations and underscore the need to correct information asymmetry and misconceptions to enhance HPV vaccination adoption. From this similarity, we can infer that the same problem in the Kuwait community, whether among university students or parents, a lack of knowledge is one of the obstacles that leads to decreased uptake of the HPV vaccine. On the other hand, in this quantitative study, when health professionals explain or advise parents to take the vaccine, they are more likely to have their children vaccinated.

Jordanian female undergraduates at health schools had a relatively high level of knowledge and attitudes toward HPV vaccination [24]. Their intention to vaccinate was derived directly from perceived susceptibility, perceived benefit, perceived barriers, and cues to action, which were consistent with the HBM. For example, in the same study, the participants explained the perceived severity of the vaccine. The study emphasized how the COVID-19 pandemic could have heightened concerns about vaccine safety and, in turn, further fueled vaccine hesitancy. Because of the high level of knowledge in Jordanian female undergraduates, which is different from our study, and the same intention regarding perceived severity in our study, even though there are several common characteristics between the 2 different countries, as both are Arabic countries, there are similarities and differences in HPV knowledge and perceived severity among different populations, respectively [24].

However, similar frameworks to explore parents’ perspectives on HPV vaccination. For instance, one study found that Arabic-speaking mothers in Australia had low understanding and awareness of HPV and its vaccine, and that cultural and religious beliefs, perceived severity, perceived benefits, perceived barriers, self-efficacy, and social norms influenced their acceptance of the vaccine [25]. For example, results found that cost, safety concerns, negative feelings, and knowledge gaps were the most common reasons given for the decline in HPV vaccine acceptability [25]. This explains that even some Arab participants living in other communities have the same perceived barriers in our study regarding the HPV vaccine, as well as low levels of knowledge. This means stigma, Kuwaiti culture is a key barrier, and low levels of expertise are similar for other Arabic participants, even in different communities.

Other studies found that Syrian mothers had low knowledge and negative attitudes toward HPV vaccination for their teen girls. Their main barriers were a lack of information, fear of side effects, and distrust of health authorities [26]. Also, the Syrian mothers are similar to the sociocultural context of the Kuwait mother community, sharing the same language and religion, which explains why the lack of knowledge and cultural barriers should be addressed to increase uptake of the HPV vaccine [26].

To provide a wider explanation regarding HBM in different areas of Middle Eastern culture and community, one study in Thailand showed Thai parents had a limited to moderate understanding of HPV and the HPV vaccination, and their acceptance of the vaccine was significantly influenced by perceived susceptibility, severity, advantages, hurdles, provocations, and values. The findings, along with similar research, suggest that parents’ understanding, views, and preferences regarding HPV vaccination for their children vary significantly across nations and cultures. This necessitates treatments customized to the needs, interests, and cultural attributes of specific people [27]. That means, in some studies across different community cultures, we can interpret that when parents have high knowledge, they will have a high degree of understanding of HBM concepts in various ways, such as through interventions from the MOH or recommendations from health professionals. So, immunization in any community will increase directly because all parents will gain more information about the perceived benefits, severity, and other HBM concepts regarding the HPV vaccine, which will ultimately lead to herd immunity. One of the limitations of this study is that it was conducted only in government schools and did not collect data from private schools, with most of the students being non-Kuwaiti citizens. Therefore, these results are unlikely, given the knowledge and health beliefs of all parents in Kuwait.

### Conclusions

Our study shares similarities and differences with other research that used the HBM or similar frameworks to investigate parents’ knowledge, beliefs, and acceptance of the HPV vaccine for their children in various countries and cultures. One notable similarity is the consistent finding that parents’ knowledge of HPV and the HPV vaccine tends to be low. In addition, the higher the knowledge level, the greater the perceived susceptibility, severity, benefits, and acceptance of the vaccine. Such a correlation highlights the need for interventions to build parents’ knowledge and awareness about HPV and the HPV vaccine everywhere because it is a crucial component of improving vaccine acceptance and absorption. Based on our findings, some preliminary conclusions and implications for health promotion and policy formulation regarding HPV vaccination in Kuwait. To start with, it is crucial to address parents’ lack of understanding of HPV and the HPV vaccine, especially among the less educated, lower-income, and Kuwaiti nationals. Comprehensive and culturally appropriate education efforts should be undertaken through schools, physicians, the media, and community organizations to create awareness and foster a better understanding of HPV and the importance of vaccination.

## Supplementary material

10.2196/75818 Multimedia Appendix 1 Descriptive for total susceptibility score for participants (male/female).

10.2196/75818 Multimedia Appendix 2 Responses to perceived susceptibility to HPV infection and related conditions.

10.2196/75818 Multimedia Appendix 3 Descriptive for total severity score for participants (male and female).

10.2196/75818 Multimedia Appendix 4 Response to the perceived severity of infection of HPV and associated conditions.

10.2196/75818 Multimedia Appendix 5 Descriptive total benefit score for participants (male and female).

10.2196/75818 Multimedia Appendix 6 Descriptive statistics for perceived benefits items.

10.2196/75818 Multimedia Appendix 7 Difference between male and female participants’ benefit score.

10.2196/75818 Multimedia Appendix 8 Descriptive for total barrier score for participants (male and female).

10.2196/75818 Multimedia Appendix 9 Descriptive statistics for perceived barrier items.

10.2196/75818 Multimedia Appendix 10 Descriptive for total self-efficacy score for participants (male and female).

10.2196/75818 Multimedia Appendix 11 Descriptive statistics for perceived self-efficacy items.

10.2196/75818 Multimedia Appendix 12 Descriptive for total cue to action score for participants (male and female).

10.2196/75818 Multimedia Appendix 13 Descriptive for cue to action items.

## Abbreviations

CC: cervical cancer
HBM: health belief model
HPV: human papillomavirus
OR: odds ratio
