# Supplementary material for: Using the Health Belief Model to Examine Parental Knowledge and Health Beliefs About Human Papilloma Virus (HPV) and iHPV Vaccine in Kuwait: Cross-Sectional Survey Study
Source: JMIR Public Health Surveill. 2025 Dec 9;11:e75818. doi: 10.2196/75818 (PMC12690283; doi:10.2196/75818)
Supplement: Multimedia Appendix 1 [file publichealth-v11-e75818-s001.docx]

| **Relationship of Respondent and the Eligible Child** | **N** | **Mean** | **Standard Deviation** | **Standard Error of Mean** |
| --- | --- | --- | --- | --- |
| Mothers’- female guardians | 363 | 28.9008 | 3.76757 | .19775 |
| Fathers’ -male guardians | 171 | 29.3860 | 5.11085 | .39084 |
| **Total** | **534** | **29.0562** | **4.24536** | **.18371** |
