# Supplementary material for: Using the Health Belief Model to Examine Parental Knowledge and Health Beliefs About Human Papilloma Virus (HPV) and iHPV Vaccine in Kuwait: Cross-Sectional Survey Study
Source: JMIR Public Health Surveill. 2025 Dec 9;11:e75818. doi: 10.2196/75818 (PMC12690283; doi:10.2196/75818)
Supplement: Multimedia Appendix 2 [file publichealth-v11-e75818-s002.docx]

| Items of Perceived Susceptibility to HPV | Overall (n=534) | Male  (n = 171) | Female (n = 363) | p-value **-A** |
| --- | --- | --- | --- | --- |
|  | Yes (%) | Yes (%) | Yes (%) |  |
| I am at risk of HPV infection | 120 (22.5) | 46 (26.9) | 74 (20.4) | 0.116 |
| My daughter/son is at risk of contracting HPV | 119 (22.3) | 53 (31.0) | 66 (18.2) | **0.001*** |
| The chance that my adolescent daughter will get cervical cancer during her lifetime is high | 69 (12.9) | 24 (14.0) | 45 (12.4) | 0.698 |
| The chance that my adolescent son will get anal cancer during his lifetime is high | 64 (12.0) | 23 (13.5) | 41 (11.3) | 0.567 |
| The chance that my adolescent son will get penile cancer during his lifetime is high | 74 (13.9) | 27 (15.8) | 47 (12.9) | 0.452 |
| I think other people are more likely to contract HPV than my daughter/son | 214 (40.1) | 82 (48.0) | 132(36.4) | **0.014*** |
| I cannot get HPV-related cancer because I have a good lifestyle | 152 (28.5) | 57 (33.3) | 95 (26.2) | 0.108 |
| I worry about the diseases you can get if you get infected with HPV | 185 (34.6) | 54 (31.6) | 131(36.1) | 0.355 |
| Vaccination against HPV is only important when you become sexually active | 213 (39.9) | 84 (49.1) | 129(35.5) | **0.004*** |
| I don’t see vaccination against HPV as important. | 267 (50.0) | 88 (51.5) | 179(49.3) | 0.711 |
| **Notes:**  ***Indicates statistical significance**  **A indicates the chi-square test.** | | | | |
