# Supplementary material for: Using the Health Belief Model to Examine Parental Knowledge and Health Beliefs About Human Papilloma Virus (HPV) and iHPV Vaccine in Kuwait: Cross-Sectional Survey Study
Source: JMIR Public Health Surveill. 2025 Dec 9;11:e75818. doi: 10.2196/75818 (PMC12690283; doi:10.2196/75818)
Supplement: Multimedia Appendix 3 [file publichealth-v11-e75818-s003.docx]

| Relationship of Respondent and the eligible child | N | Mean | Standard Deviation | Standard Error of Mean |
| --- | --- | --- | --- | --- |
| Mothers’- female guardians | 363 | 30.1736 | 9.2402 | .48499 |
| Fathers’ -male guardians | 171 | 31.0529 | 9.5333 | .72904 |
| Total | **534** | **30.4551** | **9.3351** | **.40397** |
