# Supplementary material for: Using the Health Belief Model to Examine Parental Knowledge and Health Beliefs About Human Papilloma Virus (HPV) and iHPV Vaccine in Kuwait: Cross-Sectional Survey Study
Source: JMIR Public Health Surveill. 2025 Dec 9;11:e75818. doi: 10.2196/75818 (PMC12690283; doi:10.2196/75818)
Supplement: Multimedia Appendix 4 [file publichealth-v11-e75818-s004.docx]

| Items of Perceived Severity of HPV infection | Overall (n=534) | Male  (n = 171) | Female  (n = 363) | p-value **A** |
| --- | --- | --- | --- | --- |
|  | Yes (%) | Yes (%) | Yes (%) |  |
| I believe that HPV could result in severe health problems for my daughter/son | 249 (46.6) | 93 (54.4) | 156 (43.0) | **0.018*A** |
| I believe that HPV infection could lead to cancer diseases | 272 (50.9) | 95 (55.6) | 177 (48.8) | 0.17 |
| If my daughter/son was infected with HPV, it would be affects to her/his study | 239 (44.8) | 88 (51.5) | 151 (41.6) | **0.041*A** |
| If my daughter / son is infected with HPV, she will be affected by her daily life | 264 (49.4) | 99 (57.9) | 165 (45.5) | **0.01*A** |
| If my daughter/son was infected with HPV, it would be disruptive to her/his marriage | 238 (44.6) | 94 (55.0) | 144 (39.7) | **0.001*A** |
| Cervical cancer is a serious disease and worries me | 268 (50.2) | 84 (49.1) | 184 (50.7) | 0.807 |
| Anal cancer is a serious disease and worries me | 267 (50.0) | 89 (52.0) | 178 (49.0) | 0.578 |
| Penile cancer is a serious disease and worries me | 264 (49.4) | 90 (52.6) | 174 (47.9) | 0.357 |
| If a person becomes infected with HPV, it may lead to death | 145 (27.2) | 49 (28.7) | 96 (26.4) | 0.666 |
| **Notes:**  ***Indicates statistical significance**  **A indicates chi-square test.** | | | | |
