# Supplementary material for: Using the Health Belief Model to Examine Parental Knowledge and Health Beliefs About Human Papilloma Virus (HPV) and iHPV Vaccine in Kuwait: Cross-Sectional Survey Study
Source: JMIR Public Health Surveill. 2025 Dec 9;11:e75818. doi: 10.2196/75818 (PMC12690283; doi:10.2196/75818)
Supplement: Multimedia Appendix 6 [file publichealth-v11-e75818-s006.docx]

| Items of Perceived Benefit of HPV vaccination | Overall | Male respondent | Female respondent | p-value |
| --- | --- | --- | --- | --- |
|  | 534 | 171 | 363 |  |
| Preventing cancer diseases by HPV vaccination is better than curing them | 282 (51.8) | 107 (62.6) | 175 (48.2) | **0.003*** |
| If my daughter receives an HPV vaccine, it may help me stop worrying about cervical cancer for her | 232 (43.4) | 78 (45.6) | 154 (42.4) | 0.548 |
| I believe the HPV vaccine is effective in preventing HPV infection for my daughter/son | 237 (44.4) | 80 (46.8) | 157 (43.3) | 0.501 |
| The HPV vaccine is necessary to give me a feeling that my daughter/son is healthy | 246 (46.1) | 87 (50.9) | 159 (43.8) | 0.151 |
| If my son receives an HPV vaccine, it may help me stop worrying about anal cancer for him | 232 (43.4) | 86 (50.3) | 146 (40.2) | **0.036*** |
| If my son receives an HPV vaccine, it may help me stop worrying about penile cancer for him | 222 (41.6) | 83 (48.5) | 139 (38.3) | **0.032*** |
| If I receive the HPV vaccine, I will not contract HP | 193 (36.1) | 72 (42.1) | 121 (33.3) | 0.061 |
| I feel the HPV vaccine is safe and prevents disease | 233 (43.6) | 86 (50.3) | 147 (40.5) | **0.042*** |
| **Notes:**  ***Indicates statistical significance**  **A indicates chi-square test** | | | | |
