# Supplementary material for: Using the Health Belief Model to Examine Parental Knowledge and Health Beliefs About Human Papilloma Virus (HPV) and iHPV Vaccine in Kuwait: Cross-Sectional Survey Study
Source: JMIR Public Health Surveill. 2025 Dec 9;11:e75818. doi: 10.2196/75818 (PMC12690283; doi:10.2196/75818)
Supplement: Multimedia Appendix 7 [file publichealth-v11-e75818-s007.docx]

|  | **t** | **df** | **Sig.(2-tailed)** | **Mean Difference** | **Std. Error Difference** | **95% Confidence Interval of Difference** | |
| --- | --- | --- | --- | --- | --- | --- | --- |
|  |  |  |  |  |  | **Lower** | **Upper** |
| Total Benefit Score | -2.045 | 532 | .041 | -1.55411 | .75980 | -3.04668 | -.06153 |
