# Supplementary material for: Using the Health Belief Model to Examine Parental Knowledge and Health Beliefs About Human Papilloma Virus (HPV) and iHPV Vaccine in Kuwait: Cross-Sectional Survey Study
Source: JMIR Public Health Surveill. 2025 Dec 9;11:e75818. doi: 10.2196/75818 (PMC12690283; doi:10.2196/75818)
Supplement: Multimedia Appendix 8 [file publichealth-v11-e75818-s008.docx]

| Relationship of Respondent and the Eligible Child | N | Mean | Standard Deviation | Standard Error of Mean |
| --- | --- | --- | --- | --- |
| Mothers’- female guardians | 363 | 36.793 | 8.784 | .461 |
| Fathers’ -male guardians | 171 | 37.842 | 7.159 | .547 |
| Total | **534** | **37.129** | **8.306** | **.359** |
