# Supplementary material for: Using the Health Belief Model to Examine Parental Knowledge and Health Beliefs About Human Papilloma Virus (HPV) and iHPV Vaccine in Kuwait: Cross-Sectional Survey Study
Source: JMIR Public Health Surveill. 2025 Dec 9;11:e75818. doi: 10.2196/75818 (PMC12690283; doi:10.2196/75818)
Supplement: Multimedia Appendix 9 [file publichealth-v11-e75818-s009.docx]

| Items of Perceived Barrier to HPV | Overall | Male respondent | Female respondent | p-value **A** |
| --- | --- | --- | --- | --- |
|  | 534 | 171 | 363 |  |
| There is a need for reassurance about the safety of the HPV vaccine | 301 (56.4) | 114 (66.7) | 187 (51.5) | **0.001*** |
| There is a need for reassurance about the effectiveness of the HPV vaccine | 283 (53.0) | 106 (62.0) | 177 (48.8) | **0.006*** |
| There is a stigma associated with receiving the HPV vaccination in Kuwaiti culture | 107 (20.0) | 32 (18.7) | 75 (20.7) | 0.683 |
| Receiving an HPV vaccine may be seen as a sign of promiscuity in Kuwaiti culture | 139 (26.0) | 49 (28.7) | 90 (24.8) | 0.399 |
| My religion does not allow me to receive the HPV vaccine | 74 (13.9) | 24 (14.0) | 50 (13.8) | 0.10 |
| I do not go for the HPV vaccination because it is not available in public hospitals | 146 (27.3) | 41 (24.0) | 105 (28.9) | 0.274 |
| I will not receive the HPV vaccination if I have to pay for it | 104 (19.5) | 37 (21.6) | 67 (18.5) | 0.454 |
| My daughter/son is afraid of needles, and so would not go for a vaccine if it were available | 97 (18.2) | 28 (16.4) | 69 (19.0) | 0.538 |
| I am worried that the HPV vaccine is not safe and could have side effects | 197 (37.0) | 67 (39.2) | 130 (35.9) | 0.526 |
| I am worried that the HPV vaccine does not prevent cancer | 179 (33.5) | 65 (38.0) | 114 (31.4) | 0.158 |
| Fear of pain may be one of my barriers to receiving an HPV vaccine | 125 (23.4) | 42 (24.6) | 83 (22.9) | 0.747 |
| If I decide to give the HPV vaccine to my daughter/son, I will not tell anyone about it. | 121 (22.7) | 42 (24.6) | 79 (21.8) | 0.542 |
| **Notes:**  ***Indicates statistical significance**  **A indicates the chi-square test** |  |  |  |  |
