# Supplementary material for: Using the Health Belief Model to Examine Parental Knowledge and Health Beliefs About Human Papilloma Virus (HPV) and iHPV Vaccine in Kuwait: Cross-Sectional Survey Study
Source: JMIR Public Health Surveill. 2025 Dec 9;11:e75818. doi: 10.2196/75818 (PMC12690283; doi:10.2196/75818)
Supplement: Multimedia Appendix 10 [file publichealth-v11-e75818-s010.docx]

| Relationship of Respondent and the Eligible Child | N | Mean | Standard Deviation | Standard Error of Mean |
| --- | --- | --- | --- | --- |
| Mothers- female guardians | 363 | 22.771 | 6.208 | .325 |
| Fathers -male guardians | 171 | 25.093 | 5.319 | .406 |
| Total | **534** | **23.515** | **6.031** | **.261** |
