# Supplementary material for: Using the Health Belief Model to Examine Parental Knowledge and Health Beliefs About Human Papilloma Virus (HPV) and iHPV Vaccine in Kuwait: Cross-Sectional Survey Study
Source: JMIR Public Health Surveill. 2025 Dec 9;11:e75818. doi: 10.2196/75818 (PMC12690283; doi:10.2196/75818)
Supplement: Multimedia Appendix 11 [file publichealth-v11-e75818-s011.docx]

| Items of Perceived Self-Efficacy of HPV Vaccine | Overall (n=534) | Male (n=171) | Female (n=363) | p-value **A** |
| --- | --- | --- | --- | --- |
| It would be easy for me to encourage my daughter/son to talk to a doctor about the HPV vaccine | 251 (47.0) | 98 (57.3) | 153 (42.1) | **0.001*** |
| I feel I have good enough knowledge to make a decision about the HPV vaccine for my daughter/son | 180 (33.7) | 62 (36.3) | 118 (32.5) | 0.449 |
| I have full control over giving my daughter an HPV vaccine against cervical cancer | 226 (42.3) | 96 (56.1) | 130 (35.8) | **<0.001*** |
| I have full control over giving my son an HPV vaccine against anal cancer | 219 (41.0) | 94 (55.0) | 125 (34.4) | **<0.001*** |
| I have full control over giving my son an HPV vaccine against penile cancer | 223 (41.8) | 93 (54.4) | 130 (35.8) | **<0.001*** |
| I am confident that I could agree to the HPV vaccine if I want to for my child | 249 (46.6) | 94 (55.0) | 155 (42.7) | **0.01*** |
| I myself can do something about my health by getting vaccinated against HPV | 210 (39.3) | 88 (51.5) | 122 (33.6) | **<0.001*** |
| **Notes:**  ***Indicates statistical significance**  **A indicates the chi-square test** | | | | |
