# Supplementary material for: Using the Health Belief Model to Examine Parental Knowledge and Health Beliefs About Human Papilloma Virus (HPV) and iHPV Vaccine in Kuwait: Cross-Sectional Survey Study
Source: JMIR Public Health Surveill. 2025 Dec 9;11:e75818. doi: 10.2196/75818 (PMC12690283; doi:10.2196/75818)
Supplement: Multimedia Appendix 12 [file publichealth-v11-e75818-s012.docx]

| Relationship of Respondent and the Eligible Child | N | Mean | Standard Deviation | Standard Error of Mean |
| --- | --- | --- | --- | --- |
| Mothers’- female guardians | 363 | 23.931 | 7.072 | .3712 |
| Fathers’ -male guardians | 171 | 22.093 | 7.318 | .559 |
| Total | **534** | **23.342** | **7.196** | **.3114** |
