# Supplementary material for: Using the Health Belief Model to Examine Parental Knowledge and Health Beliefs About Human Papilloma Virus (HPV) and iHPV Vaccine in Kuwait: Cross-Sectional Survey Study
Source: JMIR Public Health Surveill. 2025 Dec 9;11:e75818. doi: 10.2196/75818 (PMC12690283; doi:10.2196/75818)
Supplement: Multimedia Appendix 13 [file publichealth-v11-e75818-s013.docx]

| **Items of Cue to Action towards HPV vaccination** | **Overall (n=534)** | **Male participants (n=171)** | **Female participants (n=363)** | **p-value A** |
| --- | --- | --- | --- | --- |
| If health professionals recommended giving my daughter/son an HPV vaccine, she/he would be vaccinated | 270(50.6) | 86 (50.3) | 184 (50.7) | 1.00 |
| Watching media reports from the MOH promoting HPV vaccines for girls and boys would encourage me to give my daughter/son the HPV vaccine | 238(44.6) | 75 (43.9) | 163 (44.9) | 0.894 |
| If a relative recommended giving my daughter/son the HPV vaccine, I would be more likely to give it to her/him | 185(34.6) | 51 (29.8) | 134 (36.9) | 0.131 |
| If any friends or relatives I know went for the HPV vaccine, I would be more likely to give it to my daughter/son as well | 189(35.4) | 50 (29.2) | 139 (38.3) | **0.052*** |
| If the MOH started to vaccinate students in school, I would agree to vaccinate my daughter/son | 248 (46.4) | 63 (36.8) | 185 (51.0) | **0.003*** |
| If there were a campaign promoting the HPV vaccine, I would agree to vaccinate my daughter/ son | 237 (44.4) | 64 (37.4) | 173 (47.7) | **0.033*** |
| If there were a campaign promoting the HPV vaccine, I would advise my relatives to vaccinate their children | 228 (42.7) | 59 (34.5) | 169 (46.6) | **0.011*** |
| **Notes:**  ***Indicates statistical significance**  **A indicates chi-square test** | | | | |
